# Biomarker Profiles Associated with COVID-19 Severity and Mortality

**DOI:** 10.3390/cimb45030128

**Published:** 2023-03-01

**Authors:** Silvia Sánchez-Díez, Carlos Gómez-Ollés, María-Jesús Cruz, Miquel de Homdedeu, David Espejo, Jaume Ferrer, Oriol Roca, Andrés Pacheco, Xavier Muñoz

**Affiliations:** 1 Pulmonology Service, Department of Medicine, Vall d’Hebron University Hospital, Autonomous University of Barcelona, 08035 Barcelona, Spain; 2 CIBER of Respiratory Diseases (CIBERES), 28029 Madrid, Spain; 3 Intensive Medicine, Vall d’Hebron University Hospital, Autonomous University of Barcelona, Barcelona 08035, Spain; 4 Department of Cell Biology and Physiology and Immunology, Autonomous University of Barcelona, 08193 Barcelona, Spain

**Keywords:** COVID-19, cytokines, serum, lung fibrosis

## Abstract

Introduction: The aim of this study was to analyze biomarkers that might predict the severity and progression of the SARS-CoV-2 infection, both in the acute phase and after recovery. Methods: Unvaccinated patients infected with the original strain of COVID-19 requiring ward (Group 1, n = 48) or ICU (Group 2, n = 41) admission were included. At the time of admission (visit 1), a clinical history was acquired, and blood samples were obtained. One and six months after discharge from the hospital (visits 2 and 3, respectively), a clinical history, lung function tests, and blood samples were carried out. At visit 2, patients also underwent a chest CT scan. Different cytokines (IL-1β, IL-2, IL-4, IL-5, IL-6, IL-7, IL-8, IL-10, IL-12p70, IL-13, IL-17A, G-CSF, GM-CSF, IFN-ɣ, MCP-1, MIP-1β, and TNF-α) and lung fibrosis biomarkers (YKL-40 and KL-6) were measured in blood samples obtained at visits 1, 2, and 3. Results: At visit 1, IL-4, IL-5, and IL-6 levels were higher in Group 2 (*p* = 0.039, 0.011, and 0.045, respectively), and IL-17 and IL-8 levels were higher in Group 1 (*p* = 0.026 and 0.001, respectively). The number of patients in Groups 1 and 2 who died during hospitalization was 8 and 11, respectively. YKL-40 and KL-6 levels were higher in patients who died. Serum YKL-40 and KL-6 levels determined at visit 2 correlated negatively with FVC (*p* = 0.022 and *p* = 0.024, respectively) and FEV1 (*p* = 0.012 and *p* = 0.032, respectively) measured at visit 3. KL-6 levels also correlated negatively with the diffusing capacity of the lungs for carbon monoxide (DLCO, *p* = 0.001). Conclusions: Patients who required ICU admission had higher levels of Th2 cytokines, while patients admitted to the ward showed an innate immune response activation, with IL-8 release and Th1/Th17 lymphocyte contribution. Increased levels of YKL-40 and KL-6 were associated with mortality in COVID-19 patients.

## 1. Introduction

The disease caused by the severe acute respiratory syndrome (SARS)-CoV-2 coronavirus has affected millions of people worldwide since its outbreak in 2019, and the severity of the disease differs from person to person. After SARS-CoV-2 infection, the first variants of the virus incubate in the upper respiratory tract for 2–5 days before the development of mild clinical symptoms, such as dry cough and throat pain [1]. At this stage, the innate immune response is limited, unless there is a significant increase in the viral load. Viral progression in the airways is related to a more robust immune response and induces the appearance of other symptoms, such as muscle aches, fever, and in the worst cases, breathing difficulty [2]. In approximately 20% of non-vaccinated patients, there is alveolar progression with pulmonary infiltrates and severe disease, which is often associated with an excessive inflammatory response. At this point, patients require hospitalization due to the appearance of pneumonia and low oxygen saturation in the blood [3].

According to the literature, severe SARS-CoV-2 can trigger an excessive immune response, with massive production of inflammatory cytokines and chemical mediators, known as the cytokine storm [4,5,6,7]. Cytokine levels rise during the systemic inflammatory response syndrome and may lead to multi-organ failure in some individuals. Severe COVID-19 patients also present high neutrophil counts and low numbers of lymphocytes in the blood. Moreover, CD8+T cells in COVID-19 patients appear to be functionally exhausted, indicated by a lower production of IFN-ɣ, TNF-α and IL-2 [8,9]. Recent findings have also highlighted that toll-like receptors (TLR) bind to SARS-CoV-2, inducing the formation and release of pro-inflammatory cytokines such as pro-IL-1β, which later becomes IL-1β, causing lung inflammation and fibrosis [10,11].

While our understanding of the acute phase of the disease has expanded rapidly, little is known about the consequences of COVID-19 following clinical remission, although it is assumed that these consequences may be related to events in the acute phase. In addition, it is unclear whether (and if so, how) the profiling of cytokines can be used as a prognostic biomarker for disease outcome and control. Some patients successfully overcome acute COVID-19 infection, without any sequelae. However, patients who are more severely ill can present chronic respiratory symptoms and abnormal chest imaging manifestations after discharge from the hospital [12,13], in some cases associated with a proliferative response characterized by tissue repair that may progress to lung fibrosis [14]. 

A deeper understanding of the inflammatory and immunological patterns in post-COVID-19 pneumonia is necessary to be able to design follow-up strategies for patients recovering from acute disease, both in the hospital and after discharge. In this context, the present study assesses possible biomarkers of innate and adaptive immunity that can predict the severity and progression of COVID-19 disease towards stages of respiratory sequelae developed in unvaccinated patients infected with the original strain of COVID-19.

## 2. Material and Methods

### 2.1. Study Population

**Figure 1:**
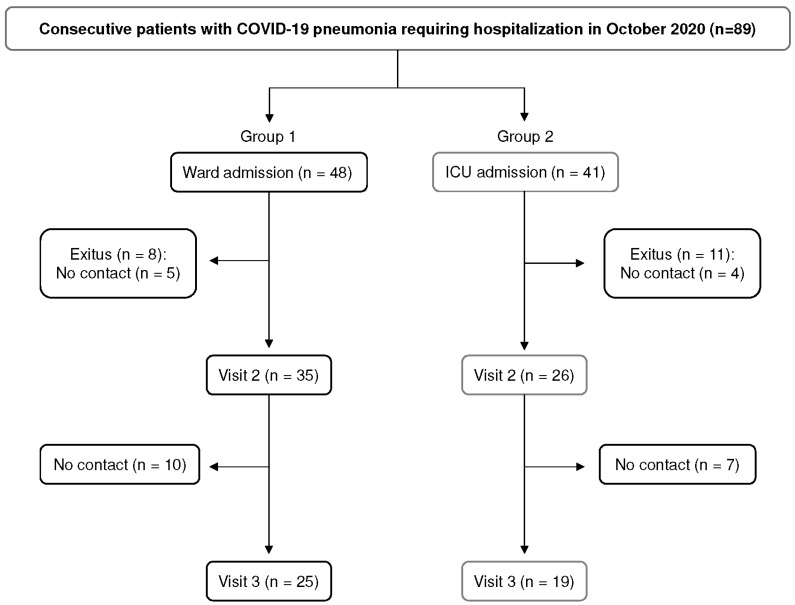
Flow chart of patient enrollment. A total of 89 consecutive unvaccinated patients with pneumonia due to the original strain of COVID-19 diagnosed at Vall d’Hebron University Hospital (Barcelona, Spain) in October 2020 requiring ward (Group 1, n = 48) or intensive care unit (ICU) admission (Group 2, n = 41) were included in the study. A total of 8 patients from Group 1 and 11 from Group 2 died during hospitalization due to COVID-19 infection. Moreover, 5 patients from Group 1 and 4 from Group 2 could not be contacted for the second visit of follow-up. At visit 3, 10 patients from Group 1 and 7 from Group 2 could not be contacted.

We performed a prospective study using a representative sample of 89 consecutive patients over the age of 18 with pneumonia due to COVID-19 diagnosed at our center (Vall d’Hebron University Hospital, Barcelona, Spain) and requiring ward (Group 1, n = 48) or intensive care unit (ICU) admission (Group 2, n = 41) in October 2020 (rate of ward admission: 12 patients/day; rate of ICU admission: 5 patients/day) (Figure 1). At the time of admission (visit 1), data from the clinical history were recorded (sociodemographic data, comorbidities, and ventilator support), and an X-ray was performed to confirm the diagnosis of pneumonia. At this stage of the disease, almost all patients were treated with dexamethasone. One and six months after discharge from the hospital (visits 2 and 3, respectively), patients were seen at the pulmonology service of our center and underwent a more exhaustive clinical history, including lung function tests. At visit 2, patients also underwent a high-resolution computed tomography (HRCT) chest scan, and at visit 3, this imaging test was only performed in patients with abnormalities upon physical examination and/or respiratory function tests compatible with respiratory sequelae. At visit 1, 2, and 3, blood samples were obtained, and after blood centrifugation (3000 rpm during 10 min), serum samples were obtained and stored at −80 °C for subsequent biomarker analysis.

The study was approved by the local ethics committee (Hospital Vall d’Hebron Ethics Committee approval PR(AG)219/2020), and all subjects signed an informed consent document prior to their participation.

### 2.2. Pulmonary Function Testing

A complete pulmonary function study, including forced spirometry and diffusion capacity of the lung for carbon monoxide (DLCO), was carried out in all participants during follow-up on a MasterLab system (MasterLab, Jaeger, Germany), in accordance with the guidelines of the European Respiratory Society and American Thoracic Society [15]. All data were expressed as percentages of published predicted values. The theoretical values were the ones proposed by the Global Lung Function Initiative (GLI) [16]. Forced vital capacity (FVC), forced expiratory volume in 1 second (FEV1), FEV1/FVC ratio, and DLCO were considered low at values of <80%, 80%, 70%, and 80% of the predicted value, respectively.

### 2.3. Chest HRCT Scanning

Chest high-resolution computed tomography was performed with 1 mm cuts at 10 mm intervals in maximum inspiration during follow-up. All the imaging features were interpreted by two pulmonologists, working independently. To interpret controversial images, an expert radiologist was consulted. High-resolution computed tomography scans were evaluated for the following characteristics: location (unilateral or bilateral) and distribution (central, peripheral, or both) of the lesion, type of lesion (simple, multiple, or diffuse), presence of ground glass, consolidation, linear opacities, reticulation, and/or mixed pattern, and interstitial changes.

### 2.4. Cytokine Analysis

The concentrations of 17 cytokines/chemokines, including Th1, Th2, and Th17 cytokines, and pro-inflammatory cytokines associated with infectious diseases and inflammation were measured: IL-1β, IL-2, IL-4, IL-5, IL-6, IL-7, IL-8, IL-10, IL-12p70, IL-13, IL-17A, G-CSF, GM-CSF, IFN-ɣ, MCP-1, MIP-1β, and TNF-α. Cytokines were measured in serum samples obtained at each visit using human cytokine multiplex magnetic bead panels (Bio-Plex Pro Human Cytokine 17-plex Assay, Bio-Rad Laboratories S.A., Madrid, Spain), according to the manufacturer’s instructions. Lower limits of detection were 0.24, 0.75, 0.04, 0.86, 0.34, 1.22, 0.36, 0.69, 0.78, 0.22, 1.16, 3.63, 0.19, 1.05, 0.44, 1.41, and 1.13 pg/mL for IL-1β, IL-2, IL-4, IL-5, IL-6, IL-7, IL-8, IL-10, IL-12p70, IL-13, IL-17A, G-CSF, GM-CSF, IFN-ɣ, MCP-1, MIP-1β, and TNF-α, respectively.

### 2.5. YKL-40 and KL-6 Analysis

YKL-40 levels were measured in serum samples obtained at each visit by an YKL-40 enzyme immunoassay kit (Quidel, San Diego, CA, USA), in accordance with the manufacturer’s instructions.

KL-6 levels were measured in serum samples obtained at each visit by a chemiluminescent enzyme immunoassay (Lumipulse G KL-6, Fujirebio Iberia SL, Barcelona, Spain) using the Lumipulse G System and in accordance with the manufacturer’s instructions.

### 2.6. Statistical Analysis

Categorical variables are expressed as absolute numbers and their corresponding percentages, and continuous variables as means (standard deviation) or medians (range), depending on the symmetry of their distribution. Comparisons between categorical variables were performed using Pearson’s Chi-square test or Fisher’s exact test, according to data distribution evaluated with the Shapiro–Wilk test. Continuous variables were compared using the Student’s t-test or the Mann–Whitney test (when comparing different groups), or the paired t-test or Wilcoxon test (when comparing results in the same group at different time points), depending on data distribution. Multiple comparisons between groups were performed using one-way ANOVA or the Kruskal–Wallis test, as appropriate. Correlations between variables were calculated with the Pearson correlation coefficient (r), according to data distribution. Analyses were conducted using GraphPad Prism 6 for Windows (version 6.01, GraphPad Software Inc, San Diego, CA, USA) and IBM SPSS Statistics (version 26, IBM Corporation, Armonk, NY, USA). Differences with a *p*-value < 0.05 (two-tailed) were considered to be significant.

## 3. Results

### 3.1. Study Population

Demographic and clinical characteristics of the subjects are shown in Table 1. In Group 2, 27% of patients were active smokers compared to 8% in Group 1 (*p* = 0.025). The mean BMI for patients in Groups 1 and 2 was 30 and 33, respectively. A total of 18 patients in each group (44% Group 1, 38% Group 2) presented with obesity (BMI > 30). Respiratory comorbidities did not appear to be a risk factor for the development of COVID-19 (Table 1). The most frequent non-respiratory comorbidities in both groups were arterial hypertension, obesity, and diabetes, without significant differences between groups. A significant difference was observed in solid cancer as a non-respiratory comorbidity in patients admitted to the ward (Group 1, *p* = 0.009), and 46% of them were already cured from the neoplasia before the infection. A total of 45% of patients in Group 1 and all patients in Group 2 required ventilatory support; however, a larger number of patients admitted to the ICU (Group 2) required invasive mechanical ventilation (*p* = 0.005).

A total of 8 patients from Group 1 and 11 from Group 2 died during hospitalization due to COVID-19 infection. Moreover, 5 patients from Group 1 and 4 from Group 2 could not be contacted during follow-up (Figure 1). Lung function and radiological characteristics during follow-up are shown in Table 2. DLCO values decreased significantly in Group 2 between visits 2 and 3 (*p* = 0.044). In Group 1, HRCT scan findings were pathological in 85% of patients at visit 2 and in 90% at visit 3, compared with 95% at visit 2 and 88% at visit 3 in Group 2. At visits 2 and 3, bilateral involvement was observed in almost all patients, with lesions mostly distributed in peripheral areas. Diffuse injury, mixed pattern, and/or ground glass opacities were the most common findings in both groups at follow-up visits. The rate of ground glass opacities in Group 2 rose from 1 (5%) at visit 2 to 5 (71%) at visit 3 (*p* = 0.002). Interstitial changes were observed at visit 2 in 6 (21%) patients in Group 1 and in 10 (53%) in Group 2 (*p* = 0.034). A radiological improvement was detected in patients in both groups at visit 3.

### 3.2. Cytokine Expression Profiles

**Figure 2:**
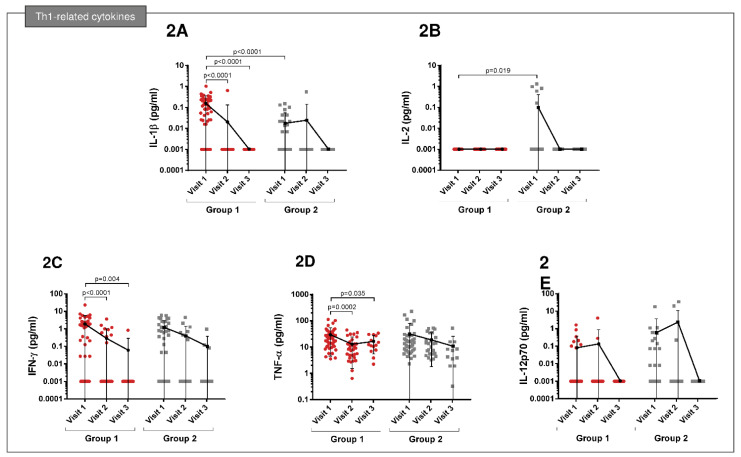
Comparison of Th1-related cytokines between patients of Group 1 and 2 from visits 1 to 3. (**A**) At visit 1, increased IL-1β levels in both groups, which were higher in patients in Group 1 than Group 2 (*p* < 0.0001). (**B**) Group 2: increased levels of IL-2 during admission (*p* = 0.019). (**C**,**D**) Increased levels of IFN-ɣ and TNF-α at visit 1 in both groups. These levels had fallen at visit 2 in patients from Group 1 (*p* < 0.0001 and 0.0002, respectively), while in Group 2, they remained high at visit 3. (**E**) No significant differences were observed for IL-12p70.

At visit 1, increased IL-1β levels were observed in both groups, and they were higher in patients in Group 1 than in Group 2 (*p* < 0.0001). At follow-up visits, this cytokine was not detectable in either group (Figure 2A). In patients admitted to the ICU (group 2), increased levels of IL-2 were found during admission (*p* = 0.019), but these levels were no longer detectable at follow-up visits (Figure 2B). Increased levels of IFN-ɣ and TNF-α were reported during infection (visit 1) in both groups. These levels had fallen at visit 2 in patients of Group 1 (*p* < 0.0001 and 0.0002, respectively), while in Group 2, they remained high at visit 3 (Figure 2C,D). No significant differences were observed in either group for IL-12p70 (Figure 2E).

**Figure 3:**
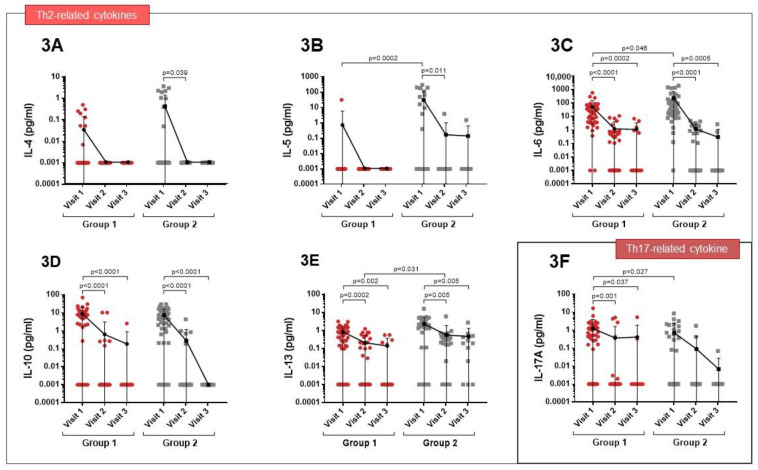
Comparison of Th2 and Th17 cytokine expression profiles between patients of Group 1 and 2 at visit 1 and in the follow-up visits. (**A**,**B**) At visit 1, increased levels of IL-4 and IL-5 in patients of Group 2 but these levels had fallen at visit 2 (*p* = 0.039 and *p* =0.011 respectively). (**C**) High IL-6 levels at visit 1 in both groups, though much higher in Group 2 (*p* = 0.046). (**D**,**E**) IL-10 and IL-13 levels increased in both groups at visit 1. (**F**) IL- 17A levels elevated at visit 1, especially in Group 1 (*p* = 0.027).

At visit 1, increased levels of IL-4 and IL-5 were observed in patients who required ICU admission (Group 2), but these levels had fallen one month after the infection (visit 2) (*p* = 0.039 and *p* = 0.011, respectively) (Figure 3A,B). High IL-6 levels were observed during infection in both groups, although they were much higher in Group 2 (*p* = 0.046). However, levels of this cytokine fell during follow-up (Figure 3C). IL-10 and IL-13 levels were increased in both groups at the time of admission, although reductions in both cytokines were observed at visits 2 and 3 (Figure 3D,E). IL- 17A levels were elevated in the acute phase of the infection, especially in Group 1 (*p* = 0.027), and then fell during follow-up (Figure 3F). 

**Figure 4:**
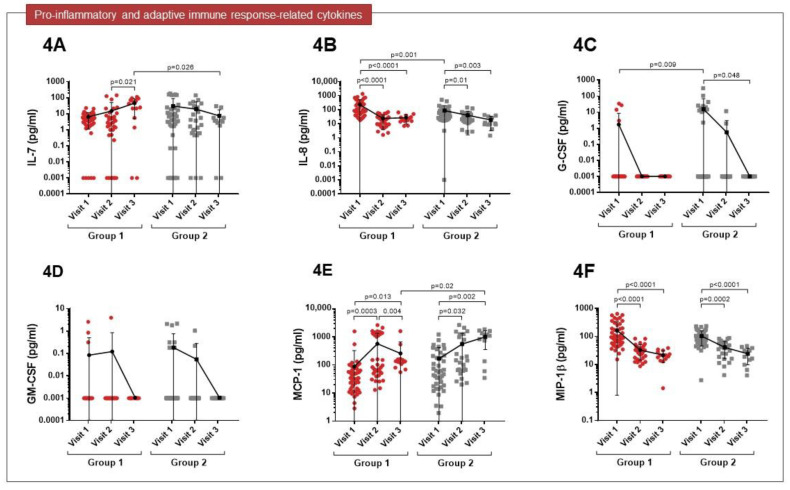
Comparison of pro-inflammatory and adaptive immune response cytokine expression profiles between patients of Groups 1 and 2 at visit 1 and at the follow-up visits. (**A**) Increased levels of IL-7 were observed in both groups at visit 3, particularly in patients of Group 1 (*p* = 0.026). (**B**) Higher levels of IL-8 at visit 1 were observed in Group 1 than in Group 2 (*p* = 0.001). (**C**) At visit 1, higher levels of G-CSF were observed in patients of Group 2 (*p* = 0.009). (**D**) No significant differences were observed in GM-CSF levels between groups. (**E**) MCP-1 levels were high in both groups at visit 2 and remained high in Group 2 at visit 3 (*p* = 0.002). (**F**) MIP-1β levels increased in both groups at visit 1.

Significantly increased levels of IL-7 were observed in both groups six months after the infection (visit 3), particularly in patients admitted to the ward (*p* = 0.026) (Figure 4A). Higher levels of IL-8 were observed during the acute phase of the disease in Group 1 than in Group 2 (*p* = 0.001), although these levels decreased with time after infection (Figure 4B). At visit 1, higher levels of G-CSF were detected in ICU patients (*p* = 0.009), but this cytokine was not detectable at visit 3 (Figure 4C). No significant differences were observed in GM-CSF levels between groups (Figure 4D). MCP-1 levels were high in both groups one month after the infection (visit 2) and remained high in Group 2 at visit 3 (*p* = 0.002) (Figure 4E). The levels of MIP-1β were increased in both groups during infection, but then fell over time (Figure 4F).

### 3.3. Levels of Pulmonary Fibrosis Biomarkers

**Figure 5:**
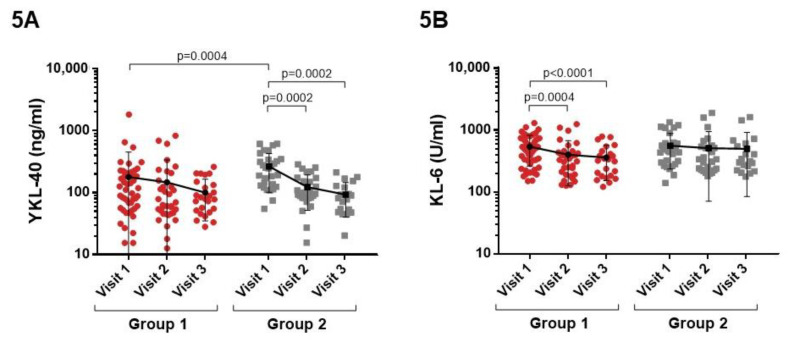
Comparison of YKL-40 and KL-6 levels between patients of Group 1 and 2 at visit 1 and in the follow-up visits (**A**,**B**). (**A**) At visit 1, higher YKL-40 levels were observed in patients of Group 2 (*p* = 0.0004). (**B**) High serum KL-6 levels were observed in both groups at visit 1, but these levels fell in patients of Group 1 in the follow-up visits.

During infection, higher YKL-40 levels were observed in patients admitted to the ICU (Group 2) (*p* = 0.0004), although these levels fell during follow-up (Figure 5A). High serum KL-6 levels were observed in both groups at the time of admission, but these levels fell in patients admitted to the ward (Group 1) in the follow-up visits (Figure 5B). 

### 3.4. Relation between Mortality and Cytokines or Pulmonary Fibrotic Biomarkers

**Figure 6:**
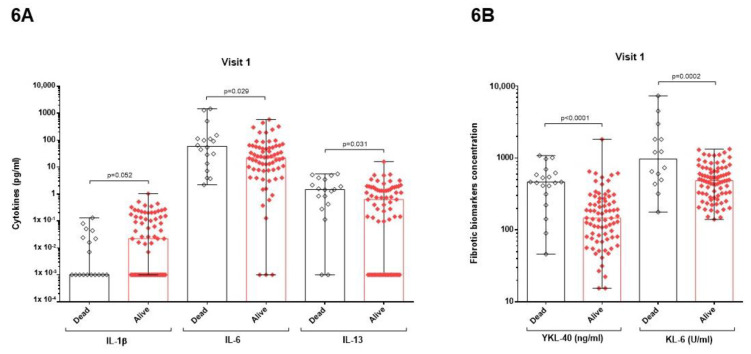
Comparison of fibrotic biomarkers (**A**) and cytokine (**B**) concentrations between patients of Group 1 and 2, according to survival or non-survival during hospitalization (visit 1). (**A**) Higher levels of IL-1β were observed in survivors (*p* = 0.052). Increased levels of IL-6 and IL-13 were related to death (*p* = 0.029 and *p* = 0.031, respectively). (**B**). Both YKL-40 and KL-6 were higher in patients who died (*p* < 0.0001 and *p* = 0.0002, respectively).

Dividing patients according to survival or non-survival during hospitalization (visit 1), survivors presented higher levels of IL-1β (*p* = 0.052) (Figure 6A). Increased levels of IL-6 and IL-13 were related to death (*p* = 0.029 and *p* = 0.031, respectively) (Figure 6A). Regarding fibrotic biomarkers, both YKL-40 and KL-6 were higher in patients who died (*p* < 0.0001 and *p* = 0.0002, respectively) (Figure 6B). 

### 3.5. Predictive Value of Cytokines and Pulmonary Fibrotic Biomarkers

**Figure 7:**
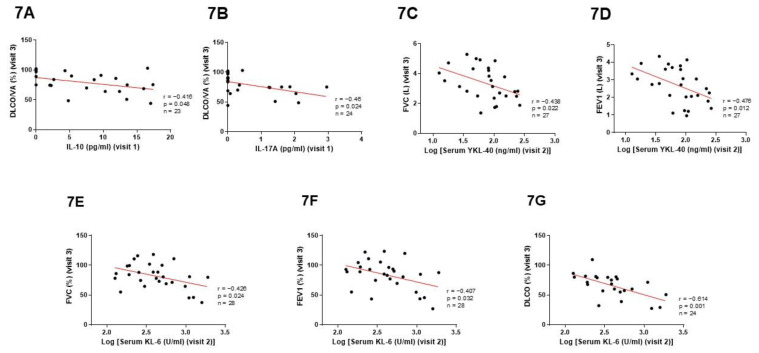
Correlations between cytokines (analyzed at visit 1) or fibrotic biomarkers (determined at visit 2) and lung function parameters (measured at visit 3). (**A**,**B**) IL-10 and IL-17A levels at visit 1 correlated negatively with DLCO/VA measured at visit 3. (% predicted, IL-10: r = −0.416, *p* = 0.048; IL-17A: r = −0.46, *p* = 0.024). (**C**–**F**) Serum YKL-40 and KL-6 levels measured at visit 2 correlated negatively with FVC (L, YKL-40: r = −0.438, *p* = 0.022; % predicted, KL-6: r = −0.426, *p* = 0.024) and FEV1 (L, YKL-40: r = −0.476, *p* = 0.012; % predicted, KL-6: r = −0.407, *p* = 0.032) measured at visit 3. (**G**) KL-6 levels at visit 2 correlated negatively with DLCO (% predicted, r = −0.614, *p* = 0.001) measured at visit 3.

IL-10 and IL-17A levels analyzed at baseline (visit 1) correlated negatively with DLCO/VA measured six months after hospitalization (% predicted, IL-10: r = −0.416, *p* = 0.048; IL-17A: r = −0.46, *p* = 0.024) (Figure 7A and 7B, respectively).

Serum YKL-40 and KL-6 levels measured one month after the infection (visit 2) correlated negatively with FVC (L, YKL-40: r = -0.438, *p* = 0.022; % predicted, KL-6: r = −0.426, *p* = 0.024) and FEV1 (L, YKL-40: r = −0.476, *p* = 0.012; % predicted, KL-6: r = −0.407, *p* = 0.032) measured at visit 3 (Figure 7C–F). In addition, KL-6 levels at visit 2 correlated negatively with DLCO (% predicted, r = −0.614, *p* = 0.001) measured six months after recovery (Figure 7G). 

## 4. Discussion

The present study assesses the profile of Th1, Th2, Th17, pro-inflammatory cytokines, and levels of pulmonary fibrosis biomarkers in patients with COVID-19, during infection and after recovery. High concentrations of IFNɣ, TNFα, IL-10, IL-13, and MIP-1β were found during infection, as well as high concentrations of IL-7 and MCP-1 after recovery, in both the hospital ward and the ICU. However, the presence of some biomarkers differed between the groups during hospitalization. Specifically, patients who required admission to the ICU exhibited higher levels of cytokines related to a Th2-type response, such as IL-4, IL-5 and IL-6, while patients admitted to the ward presented high levels of innate response proinflammatory cytokines, such as IL-8 or IL-1β, but also IL-17A. A relationship was also observed between the markers of progression to pulmonary fibrosis (i.e., YKL-40 and KL-6), IL-6, IL-10, and IL-13 cytokines and mortality. Moreover, YKL-40, KL-6, IL-10 and IL-17 were predictors of lung function in these patients.

Recent studies have shown an exaggerated immune response in individuals with severe COVID-19 illness [17]. These patients showed high levels of inflammatory cytokines such as IL-1β, IL-2, IL-6 IL-7, IL-8, IL-10, TNF-α, and/or GM-CSF [18]. In fact, more than 20 cytokines have been linearly associated with increased viral load and lung injury, and are considered potential biomarkers for the severity of the disease. Our reports of high levels of the cytokines mentioned above in our study population are in line with those described in the literature. However, in the present study, the response differed depending on the severity of the disease. In this regard, an increase in Th1 cytokines was observed in all patients, but the patients admitted to the ICU, and who therefore, exhibited more serious disease, had increased levels of certain Th2 response cytokines.

In relation to this differential response, some studies have associated the severity of COVID-19 with a specific cytokine profile. For instance, in 41 subjects from the Wuhan region with COVID-19, Huang et al. [19] observed that infected patients presented high plasma levels of IL-1β, IFNɣ, and MCP-1, which likely leads to the activation of a Th1 cell profile. In addition, recent studies analyzing the immune response after vaccination observed that, in vaccinated patients, the response to infection is characterized by a Th1 profile that is associated with a better outcome in infected patients [20]. However, our findings highlighted that patients admitted to the ICU had a higher secretion of Th2-related cytokines (e.g., IL-4, IL-5, and IL-6). The specific activation of type 2 immunity has also been confirmed by other groups. Roncati et al. [21] reported that in all 15 peripheral blood samples analyzed from intensive care COVID-19 patients, cytological signals of a Th2 immune response were found, namely eosinophilia, basophilia, degranulated eosinophils, and plasma cells. 

In addition, after recovery from the infection, both groups of patients presented elevated levels of IL-7, a pleiotropic cytokine essential for lymphocyte survival and expansion [22]. Interestingly, IL-7 shows proven efficacy as an antiviral agent and has been shown to restore lymphocyte counts and functional activity, leading to decreased viral load and clinical improvement in several life-threating viral infections. It also increases CD4 and CD8 T-cell levels 3-fold, improves T-cell activation, and reduces levels of proinflammatory cytokines [23]. The increase observed in this cytokine may indicate a restoration of the immune system after infection. 

Pulmonary fibrosis is a known sequela of ARDS. In this context, early analyses of patients with COVID-19 at hospital discharge suggest that more than one-third of recovered patients develop fibrotic abnormalities [24]. The use of biomarkers in the diagnosis and prognosis of pulmonary fibrosis in these patients may be particularly interesting, given the difficulty of establishing a well-defined diagnosis in early stages of the disease. YKL-40, also known as chitinase 3-like-1, and Krebs von den Lungen-6 (KL-6) are two promising biomarkers that may play an important role in the diagnosis and prognosis of patients with interstitial lung diseases. YKL-40 is mainly secreted by macrophages, neutrophils, and epithelial cells, but also by fibroblasts and other cells [25,26]. Although the function of YKL-40 is still debatable, there is some evidence that it has effects on fibroblasts [26,27]. KL-6 is a human mucin primarily expressed in regenerating type II pneumocytes and the bronchiolar epithelium. This glycoprotein has chemotactic and anti-apoptotic effects on fibroblasts, promoting fibroblast proliferation and the progression of pulmonary fibrosis [28,29].

In the present study, we found a possible relationship between these two biomarkers and patient mortality. Although this is an issue that has not been studied in depth, certain authors have shown that serum concentrations of KL-6 were only elevated in severely ill patients admitted to the ICU and requiring intubation and mechanical ventilation for diffuse interstitial pneumonia, but not in mild to moderately ill patients with less severe respiratory impairment [30,31,32]. In this context, some studies published in the literature suggest that COVID-19 patients also had higher levels of YKL-40 compared to control populations and that, within the COVID-19 population, YKL-40 was an indicator of the severity of infection, since it is linked to complications such as ICU admission [33]. Our findings also demonstrate that in these patients, YKL-40 and KL-6 levels remained elevated during follow-up, and that these levels correlated with reduced lung function, especially FVC, FEV1, or DLCO six months after the infection. In fact, recent studies have demonstrated that COVID-19 infection can cause lung function impairment, manifested as restricted ventilation dysfunction, small airway dysfunction, and diffuse dysfunction [34]. Ferioli et al. [35] also reported that the most common six-month functional alteration observed after COVID-19 hospitalization was DLCO impairment, which seems to be related to the severity of acute pneumonia. In this context, a study carried out in 87 patients concluded that severely ill COVID-19 patients had higher prevalence rates of abnormal spirometry and residual fibrosis as seen in radiography when compared to patients with mild symptoms and non-severe pneumonia [36]. Thus, in these patients, special attention should be paid to the development of pulmonary fibrosis as a possible sequel to infection by the SARS-CoV-2 virus; KL-6 and YKL-40 are useful tools in this follow-up. 

In this study, an association was also observed between the prognosis of patients with COVID-19 infection and increased levels of certain cytokines. In particular, patients who did not survive hospitalization had higher levels of IL-6, IL-10, and IL-13. In this regard, in a study carried out in more than 1400 patients hospitalized with COVID-19 disease, Del Valle et al. [37] concluded that high serum IL-6 levels at the time of hospitalization were strong and independent predictors of patient death. Similarly, Laguna-Goya et al. [38] reported that increased IL-6 levels were predictive of mortality in these patients. As for IL-13, recently published studies also demonstrated the association of this cytokine with severe outcomes in patients with COVID-19 [39,40]. 

To the best of our knowledge, our study is the first to establish a negative correlation between IL-10 and IL-17 levels at the moment of the acute infection with lung function, especially DLCO/VA values, after recovery from COVID-19. However, some studies in the literature highlight the association between increased levels of IL-10 and disease severity and mortality in patients with COVID infection throughout the hospitalization period [41,42,43]. As for IL-17, there is no evidence of its relationship with pulmonary parameters in follow-up visits. Nevertheless, Maslennikov et al. [44] reported that during hospitalization, patients with COVID-10 who received IL-17 antagonist treatment presented lower mortality rates and required ICU admission and additional ventilatory support less frequently. In addition, in a study carried out in 171 patients with severe COVID-19 pneumonia, Avdeev et al. [45] concluded that anti-IL-17 therapy might mitigate the inflammatory response and improve oxygenation in these patients. 

This study has some limitations. The first is in relation to the follow-up study, as we were only able to perform a short follow-up time and to reevaluate 61 (in visit 2) and 44 (in visit 3) of the 81 patients initially included, mainly due to exitus. In addition, therapies to which patients were subjected during hospitalization should be recorded in clinical histories to evaluate their possible effects on the inflammatory response. Secondly, the small sample size in the present study makes it difficult to draw definitive conclusions. Third, about 40% of the patients in the two groups expressed a BMI value > 30 (Kg/m^2^); therefore, they are obese and probably have low-grade inflammation, with a higher expression of various pro-inflammatory cytokines, regardless of SARS-CoV-2 infection. Despite this fact, the number of obese people is similar in the two groups, so the observed differences can generally be attributed to SARS-CoV-2 infection. Moreover, as a single-center study, the study has limited external validity. Future multicenter studies are necessary to clarify the role of these cytokines in the severity of disease and mortality in COVID-19. In this sense, the study of biomarkers such as cytokines in COVID-19 patients has shed light on the pathogenesis of the infection of SARS-CoV-2. Nevertheless, molecular and cellular studies of the pathogenesis of this novel coronavirus are still in the early stages of research. For this reason, one of the lessons learned for the current infections is that further studies are needed to clarify the exact roles of cellular signaling pathways once SARS-CoV-2 infects its host cell, which will allow for the identification and design of better molecular drugs.

In conclusion, patients with severe COVID-19 pneumonia had higher levels of Th2 cytokines such as IL-5 and IL-13. This may indicate the existence of a Th2 immune response that contributes directly to the immunopathology of the infection. Patients with mild infection showed an innate immune response activation, with IL-8 release and the involvement of Th1/Th17 lymphocytes. Increased levels of IL-6, IL-13, YKL-40, and KL-6 were associated with mortality in COVID-19 patients. 

## Figures and Tables

**Table 1:** Characteristics and ventilatory support of the subjects included in the study.

	Group 1 (n = 48)	Group 2 (n = 41)	*p* Value
**Sociodemographic and clinical data**			
Age (years), median (range)	66 (25–90)	63 (40–76)	0.403
Sex (male), n (%)	31 (64)	33 (80)	0.106
BMI (Kg/m^2^), median (range)	30 (15–41)	32.9 (25–33)	0.499
BMI > 30 (Kg/m^2^), n (%)	18 (38)	18 (44)	0.665
Smoking habit, n (%)			0.041
Smoker	4 (8)	11 (27)	0.025
Former smoker	21 (44)	11 (27)	0.123
Non-smoker	21 (44)	16 (39)	0.673
Survival, n (%)	40 (83)	30 (73)	0.303
**Respiratory comorbidities, n (%)**			
Asthma	2 (4)	0 (0)	0.497
Obstructive sleep apneas	5 (10)	0 (0)	0.059
Chronic obstructive pulmonary disease	5 (10)	0 (0)	0.059
Bronchiectasis	0	1 (2)	0.461
Pulmonary arterial hypertension	2 (4)	0 (0)	0.497
Pulmonary fibrosis	1 (2)	0 (0)	1.000
Other interstitial diseases	2 (4)	0 (0)	0.497
Lung transplant	2 (4)	0 (0)	0.497
**Non-respiratory comorbidities, n (%)**			
Arterial hypertension	24 (50)	20 (49)	1.000
Obesity	18 (38)	18 (44)	0.665
Diabetes	12 (25)	7 (17)	0.441
Chronic renal insufficiency	6 (12)	3 (7)	0.498
Immunosuppression	3 (6)	2 (5)	1.000
Ischemic heart disease	7 (15)	1 (2)	0.065
Heart failure	1 (2)	0 (0)	1.000
Atrial fibrillation	3 (6)	0 (0)	0.246
Other cardiac diseases	4 (8)	2 (5)	0.683
Solid cancer	13 (27)	2 (5)	0.009
Cured	6 (46)	2 (100)	0.467
Leukemia	3 (6)	0 (0)	0.246
Psychiatric disease	5 (10)	3 (7)	0.721
Gastroesophageal reflux	0 (0)	1 (2)	1.000
**Ventilatory support, n (%)**	**(n = 47)**	**(n = 40)**	
Yes	21 (45)	40 (100)	<0.0001
**Type of support, n (%)**			
High-flow nasal cannula (HFNC)	18 (86)	26 (65)	0.133
Non-invasive mechanical ventilation (NIMV)	3 (14)	2 (5)	0.329
Invasive mechanical ventilation (IMV)	13 (62)	37 (93)	0.005
Extracorporeal membrane oxygenation	1 (5)	4 (10)	0.651
Oxygen therapy 100%	2 (10)	3 (8)	1.000

A single patient may present more than one comorbidity or ventilatory support. Percentages in each line are calculated from the total population, except for patients with a neoplasia considered cured and type of ventilatory support, where percentages are calculated from the population with solid cancer and the population with support requirements, respectively. Some variables have missing values: Group 1 (2 for smoking habit, 1 for ventilatory support); Group 2 (3 for smoking habit, 1 for ventilator support).

**Table 2:** Lung function and HRCT characteristics in the study population during follow-up.

	Group 1			Group 2		
**Pulmonary function parameters**	**Visit 2 (n = 35)**	**Visit 3 (n = 25)**	***p* Value**	**Visit 2 (n = 26)**	**Visit 3 (n = 19)**	***p* Value**
FVC (% predicted), median (range)	87 (41–118)	84 (45–118)	0.533	85 (53–137)	81 (37–111)	0.143
FVC < 85%, n (%)	13 (37)	9 (36)	1.000	10 (38)	7 (37)	1.000
FEV1 (% predicted), median (range)	91 (42–134)	89 (43–124)	0.089	91 (56–139)	88 (27–122)	0.653
FEV1 < 80%, n (%)	7 (20)	6 (24)	0.758	6 (23)	3 (16)	0.712
FEV1/FVC (% predicted), median (range)	81 (52–100)	82 (43–99)	0.109	84 (76–98)	86 (56–90)	0.25
FEV1/FVC < 70%, n (%)	3 (9)	2 (8)	1.000	0 (0)	1 (5)	0.422
DLCO (% predicted), median (range)	70 (23–94)	73 (27–86)	0.457	62 (25–106)	57 (29–110)	0.044
DLCO < 80%, n (%)	22 (63)	10 (40)	0.116	16 (62)	8 (42)	0.237
DLCO/VA (% predicted), median (range)	75 (31–123)	75 (44–100)	0.649	78 (42–109)	85 (19–103)	0.097
**HRCT, n (%)**	**Visit 2 (n = 33)**	**Visit 3 (n = 11)**	***p* Value**	**Visit 2 (n = 20)**	**Visit 3 (n = 8)**	***p* Value**
**Pathological findings**						
Yes	28 (85)	10 (90)	1.000	19 (95)	7 (88)	0.497
**Location**						
Unilateral	1 (4)	0 (0)	1.000	0 (0)	0 (0)	1.000
Bilateral	27 (96)	10 (100)	1.000	19 (100)	7 (100)	1.000
**Distribution**						
Central	1 (4)	0 (0)	1.000	0 (0)	0 (0)	1.000
Peripheral	25 (89)	10 (100)	0.552	19 (100)	7 (100)	1.000
Central and peripheral	2 (7)	0 (0)	1.000	0 (0)	0 (0)	1.000
**Type of injury**						
Simple	1 (4)	0 (0)	1.000	0 (0)	0 (0)	1.000
Multiple	1 (4)	0 (0)	1.000	0 (0)	1 (14)	0.269
Diffuse	26 (92)	10 (100)	1.000	19 (100)	6 (86)	0.269
**Main imaging features**						
Ground glass	5 (18)	4 (40)	0.205	1 (5)	5 (71)	0.002
Consolidation	2 (7)	0 (0)	1.000	0 (0)	0 (0)	1.000
Linear opacities	0 (0)	1 (10)	1.000	0 (0)	0 (0)	1.000
Reticulation	3 (11)	0 (0)	0.552	5 (26)	0 (0)	0.278
Mixed pattern	15 (54)	5 (50)	1.000	11 (58)	2 (29)	0.378
Interstitial changes	6 (21) *	0 (0)	0.168	10 (53) *	2 (29)	0. 391

Percentages in each line are calculated from the total population, except for HRCT scan parameters, where percentages are calculated from the pathological population. Some variables have missing values: Group 1 (Visit 2: 2 for FVC, 2 for FEV1, 2 for FEV1/FVC, 4 for DLCO, 4 for DLCO/VA, and 2 for HRCT; Visit 3: 8 for FVC, 8 for FEV1, 8 for FEV1/FVC, 11 for DLCO, 11 for DLCO/VA, and 14 for HRCT), and Group 2 (Visit 2: 7 for FVC, 7 for FEV1, 7 for FEV1/FVC, 7 for DLCO, 7 for DLCO/VA, and 6 for HRCT; Visit 3: 8 for FVC, 8 for FEV1, 8 for FEV1/FVC, 9 for DLCO, 9 for DLCO/VA, and 11 for HRCT). * *p* < 0.05.

## Data Availability

Clinical data is unavailable due to privacy and ethical restrictions.
